# Skin & SMAS layer remodeling technique (SSRT): Achieving both volume and lifting effects with filler treatments

**DOI:** 10.1111/srt.13809

**Published:** 2024-06-21

**Authors:** Gi‐Woong Hong, Kyu‐Ho Yi

**Affiliations:** ^1^ Samskin Plastic Surgery Clinic Seoul South Korea; ^2^ Division in Anatomy and Developmental Biology, Department of Oral Biology Human Identification Research Institute, BK21 FOUR Project, Yonsei University College of Dentistry Seoul South Korea; ^3^ Maylin Clinic (Apgujeong) Seoul South Korea

**Keywords:** aesthetic plastic surgery, dermal fillers, facial asymmetry, facial lifting, skin aging, tissue expansion

## Abstract

**Background:**

The skin & SMAS layer remodeling technique (SSRT) represents a significant advancement in facial aesthetic treatments. Traditional methods primarily address wrinkles and volume loss, whereas SSRT focuses on achieving both volume and natural lifting effects through strategic filler injections. This technique emphasizes the anatomical and dynamic properties of facial tissues, particularly the SMAS, to enhance facial symmetry and contours while maintaining natural movement.

**Materials and Methods:**

SSRT employs a combination of firm and soft hyaluronic acid fillers to sculpt a youthful, dynamic appearance, simulating the natural lift of a smiling expression. This study explores the effects of gravity on facial tissues and uses advanced imaging techniques to corroborate the shifting of tissues. The technique is compared with other recognized methods, such as true lift and myomodulation, to evaluate its effectiveness in balancing volume enhancement and dynamic facial expressions.

**Results:**

The results indicate that SSRT uniquely balances volume enhancement and dynamic facial expressions, achieving a visibly lifted but natural appearance. The use of advanced imaging techniques substantiates SSRT's effectiveness by showing the shifting of tissues. SSRT reduces the risks associated with excessive filler use and aligns with contemporary aesthetic preferences.

**Conclusion:**

SSRT offers a novel approach by mimicking the facial lift observed when lying down, providing insights into potential transformative “lifts” when upright. By strategically combining firm and soft hyaluronic acid fillers, SSRT achieves a balance of volume and lift, enhancing facial symmetry and maintaining natural movement. This technique represents a significant advancement in facial aesthetic treatments, offering a natural and dynamic alternative to traditional methods.

## 1 INTRODUCTION

With the increasing clinical application of fillers, there has been a notable shift in the methodologies used for facial rejuvenation and volumization.^1^, ^2^ Historically, fillers of varying particle sizes and consistencies were employed primarily to enhance skin quality and address complex areas such as around the eyes and mouth, particularly for various types of wrinkles.^3^, ^4^ Recent advancements in fillers characterized by high viscoelastic properties have facilitated a comprehensive approach to sculpting facial contours holistically.^5^

Globally, there is a discernible transition from traditional dermal fillers towards soft tissue volume fillers, which are now preferred for comprehensive facial contouring.^6^, ^7^ This trend is particularly evident in Asia, where surveys indicate a preference for fillers over fat grafting for enhancing facial volume broadly, rather than targeting isolated areas.^8^, ^9^, ^10^, ^11^

Achieving a harmonious and symmetrical appearance necessitates consideration of the face in motion, not merely at rest. This approach requires detailed understanding of the anatomical configuration and dynamic changes of the facial soft tissues overlying the skeletal framework, as well as the age‐related transformations these tissues undergo. An accurate facial analysis, aligned with current aesthetic ideals and personalized to individual anatomical nuances, is crucial.

The ideal facial contour, as perceived when smiling, involves a natural upward and lateral pull of the mid and lower facial tissues due to the activation of lip elevator muscles. This dynamic movement creates the illusion of a “lift”, resulting in a narrower, V‐shaped lower face with elevated, fuller anterior cheek areas. Emulating these natural soft tissue dynamics during filler application can result in a visage that appears naturally buoyant and youthful.^11^, ^12^, ^13^

Moreover, the discourse has evolved from merely augmenting facial volume to strategically achieving a lift. It is now understood that significant facial aging is not only manifested through wrinkles but also through the shifting facial contour, primarily driven by gravitational effects on increasingly lax tissues. This sagging is pronounced while upright but is altered when the orientation changes, such as lying down, as observed through 3D imaging techniques which demonstrate how tissue density increases and sags less in prone positions.^14^

Therefore, understanding the multifaceted movement and transformation of facial tissues in different orientations is imperative for achieving a truly natural lift. This perspective underpins contemporary filler techniques, such as the SSRT (Skin & SMAS layer Remodeling Technique) developed in Korea, alongside recognized methods like True Lift and Myomodulation, which all aim to harness these dynamics for enhanced facial aesthetics without adverse effects.

### 1.1 SSRT (skin & SMAS layer remodeling technique)

**FIGURE 1:**
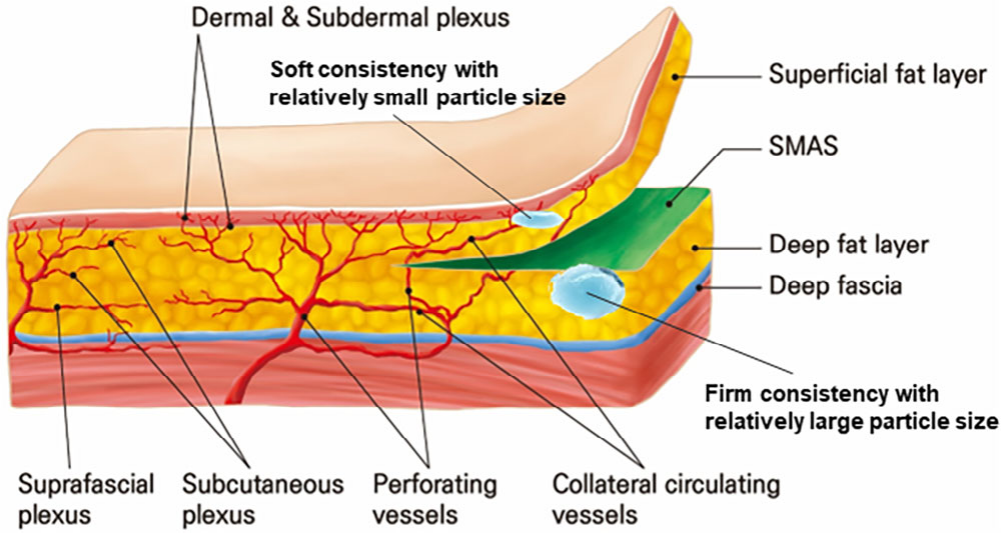
Selection of HA filler based on consistency and particle size tailored to specific facial regions.

The innovative Skin & SMAS layer Remodeling Technique (SSRT) introduced by the author represents a paradigm shift in the application of hyaluronic acid (HA) fillers, advancing beyond traditional methods primarily focused on volumizing and wrinkle reduction. This technique categorizes HA fillers into two distinct groups based on their physical properties: firm fillers characterized by their denser consistency and larger particle sizes, and soft fillers, which are more malleable and feature relatively smaller particle sizes.^15^, ^16^, ^17^ The selection criteria for these fillers extend beyond mere tactile perception; they consider the intended depth of injection, specific facial regions targeted, and the overall aesthetic objectives intended by the treatment (Figure 1).

By integrating a strategic mix of these different types of HA fillers, tailored to suit the unique requirements of various facial areas and the specific layers within the skin and subcutaneous tissues, practitioners can achieve more than just facial volumization.^18^ The technique is designed to elicit a comprehensive skin‐lifting effect, sculpting a naturally appealing V‐shaped or heart‐shaped facial contour that mimics the appearance of a smiling face. This approach not only enhances the aesthetic quality of the face but also supports dynamic expressions, contributing to a more youthful and animated appearance.

However, it's important to address misconceptions regarding the effects of filler treatments. Some critics argue that fillers merely puff up the face, creating an unnatural and balloon‐like appearance that can make the skin appear overly taut and the facial structure awkward during expressions.^19^, ^20^ While there is a kernel of truth in these concerns—particularly when excessive volumes are used—the SSRT method mitigates such outcomes by judiciously varying the consistency of the fillers used. This approach ensures that enhancements contribute to the facial structure without the drawbacks of excessive volumization, thus reducing the potential for complications associated with overuse of fillers.

**FIGURE 2:**
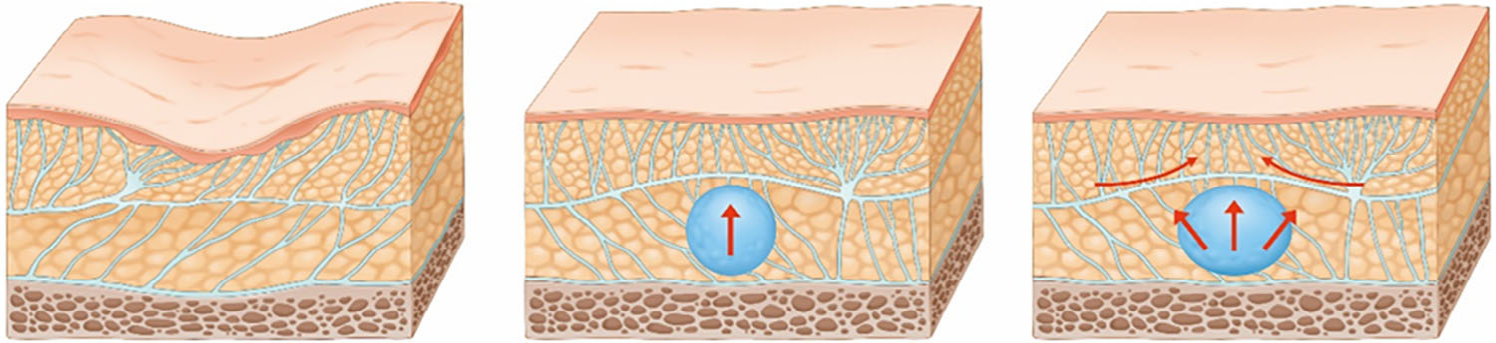
Tightening the SMAS layer creates a traction effect that extends to adjacent areas, causing these nearby SMAS layers to exhibit a tensile and lifting effect. Injected HA filler supports the SMAS layer, leading to the expansion of the injected region. This support increases the density of the subSMAS tissue, effectively tightening the SMAS layer of the injected region. The skin connected to the lifted SMAS layer exhibits the same lifting effect. Furthermore, the traction of both the skin and SMAS layer results in the compression and smoothing of adjacent bulging loose connective tissue, enhancing the overall aesthetic outcome.

At the core of the SSRT is its emphasis on the anatomical and functional aspects of the midface's SMAS layer, which plays a pivotal role in facial aesthetics.^21^ Injecting firm fillers beneath the SMAS layer increases the density within this critical area, leading to a cascade of physiological responses. As the filler expands the underlying tissues, the SMAS layer is induced to lift, exerting a tension on the adjacent connective tissues. This tension prompts the surrounding tissues to adapt, stretching and conforming to the new contours, effectively creating a lifting effect that is both natural and aesthetically pleasing (Figure 2).^19^

The SSRT, pioneered by the author, leverages a nuanced understanding of facial anatomy to achieve natural lifting effects through the strategic placement of firm fillers within the subSMAS space.^22^ This technique capitalizes on the inherent properties of the SMAS layer, which spans the entire face and integrates closely with both the skin and underlying connective tissues. When firm fillers are introduced beneath this layer, they induce a tightening effect due to their volume and density. This, in turn, exerts a mechanical pulling force on adjacent tissues, thereby fostering a lifting and volumizing outcome.

**FIGURE 3:**
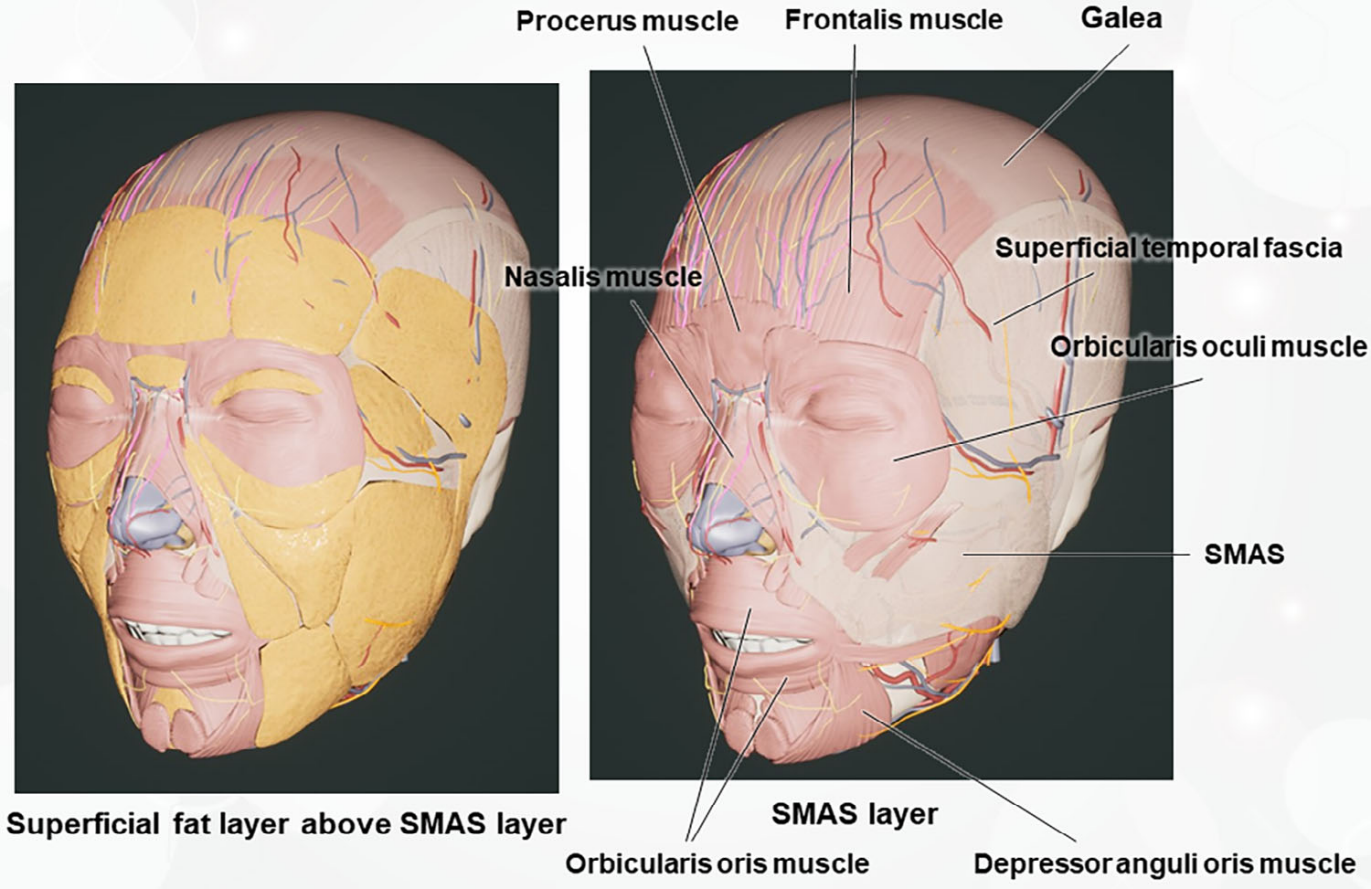
The SMAS layer extends continuously from the forehead to the neck, consisting of interconnected muscles and fascia. This anatomical structure plays a crucial role in facial aesthetics and surgical interventions, providing a foundation that influences facial contours and expressions. The seamless continuity of the SMAS allows for integrated facial movements and contributes to the uniform appearance of skin and underlying tissues across different regions of the face and neck.

Anatomical variability in the type, thickness, and density of facial areas is crucial in determining the efficacy and direction of this lifting effect.^23^ The facial structure is a complex interplay of the SMAS layer with both superficial and deep fibroadipose connective tissues, each possessing distinct mechanical properties (Figure 3). By adjusting the viscosity and cohesiveness of the fillers to suit specific facial zones, practitioners can effectively sculpt the face, enhancing both its volume and upward lift.

**FIGURE 4:**
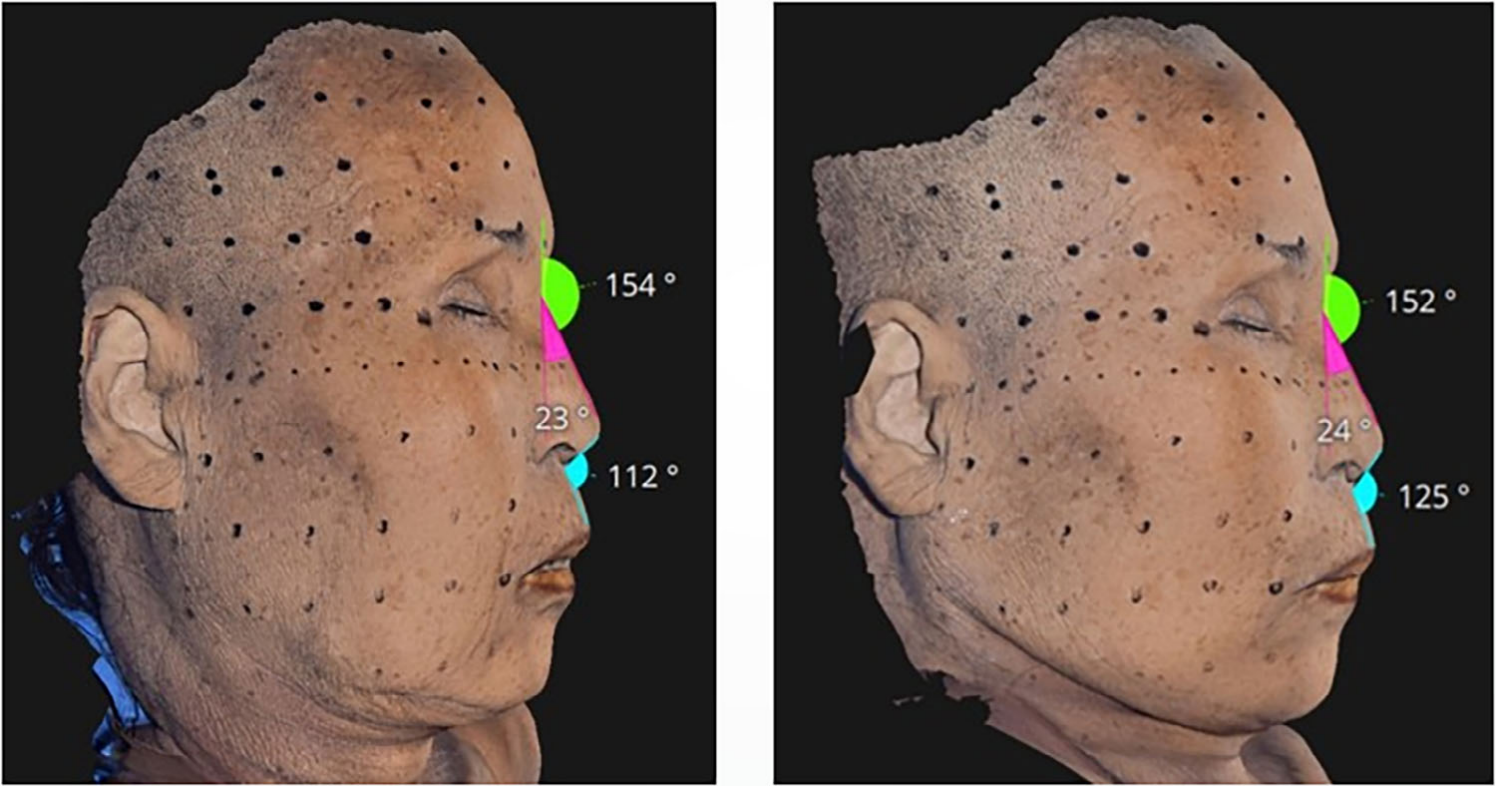
The mandible border, cheek, and perioral region undergo noticeable changes in response to positional alterations. These facial areas are highly dynamic and can shift in appearance based on various factors such as muscle movement, facial expressions, and even changes in body posture. The structural configuration of these regions is influenced by the underlying bones, muscles, and connective tissues, which adapt and react to the positional changes of the head and neck. This responsiveness highlights the complex interplay of anatomical structures that contribute to the facial aesthetics and functionality.

This lifting mechanism is contingent upon the natural tension gradients within the face.^24^ Areas of slack tissue naturally migrate towards regions where the tissues are firmer, a phenomenon amplified during aesthetic procedures. This migration mimics the gravitational effects observed when the orientation of the face shifts, such as moving from a standing to a lying position, where gravity draws the firmer tissues upward towards the scalp, thus tightening the face. Interestingly, a cadaver study illustrated that, when supine, the areas anterior to the ears and along the jawline exhibit increased tautness and definition, avoiding the accumulation of loose tissues that could otherwise create a bulging appearance along the lower facial contours (Figure 4).^25^, ^26^, ^27^

**FIGURE 5:**
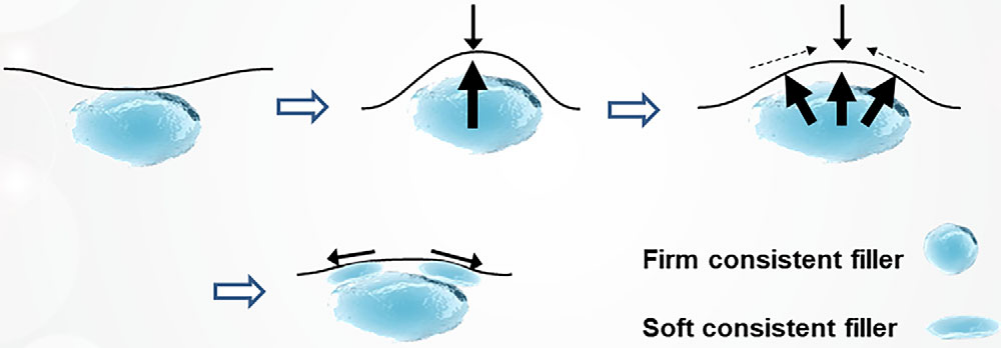
Dual plane injection techniques utilizing firm and soft consistent fillers are employed to achieve a smooth and flexible stretching surface with volumization. Firm consistent fillers are injected under the SMAS or into the deep subcutaneous layer of lubricant fibro‐adipose tissue. This approach is designed to create volume or compensate for depressed regions, providing structural support and enhancing the underlying facial contours. On the other hand, soft consistent fillers are used in the subdermal layer of superficial solid fibro‐adipose tissue or directly into the dermal layer. This method aims to create a smooth and flexible surface that can stretch and adapt to facial movements, ensuring a more natural appearance and feel to the treated areas. Together, these injection strategies integrate to form a cohesive enhancement that maintains both the aesthetic and functional properties of the facial structure.

As elucidated in relation to the facial ligamentous structures, numerous robust ligaments are present along the anterior aspects of the ears and jawline. Upon assuming a recumbent position, the more lax tissues surrounding the cheeks and oral region are observed to gravitate uniformly toward these sturdier areas, influenced by the alteration in directional forces and the inherent disparity in tissue robustness. This migration is attributed to the densification of the ligamentous tissues along the ears and jawline under gravitational influence, which results in the tightening of the adjoining skin, thus delineating a more defined jawline and concurrently exerting traction on the adjacent loose cheek and perioral soft tissues. This positional effect of reclining modifies the facial regions characterized by firm ligamentous tissues. Augmentation of these regions with a consistent, firm filler not only resolves the compacted area but also elicits traction on the neighboring skin and SMAS layer.^28^, ^29^ Such traction serves to flatten the surrounding lax soft tissues, mimicking an effect of compression. Subsequently, the administration of a soft consistent filler into the dermal and subdermal layers can ameliorate any irregularities or demarcation lines resultant from the initial firm filler injections, additionally mitigating deep wrinkles and fostering a stretching effect in zones with finer wrinkles as depicted in Figure 5.^28^, ^30^

**FIGURE 6:**
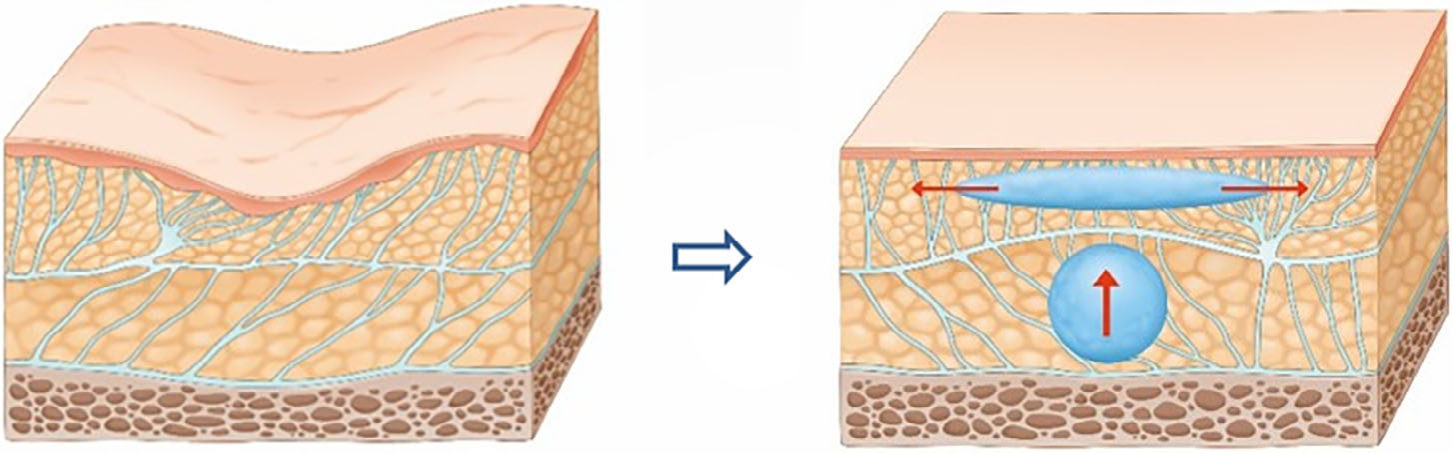
The injection of fillers into the dermal and subdermal layers significantly enhances skin aesthetics by inducing a stretching effect and increasing dermal thickness, which smooths the skin's surface and diminishes fine lines. This treatment also increases the density of dermal and subdermal tissues, improving the skin's structural integrity and reducing deformation caused by dynamic facial expressions, leading to a more youthful appearance. Additionally, filler injections at the musculocutaneous connection reduce the movement of terminal muscle fibers, stabilizing the overlaying skin and significantly improving the appearance of wrinkles and irregularities for a smoother and more even complexion.

**FIGURE 7:**
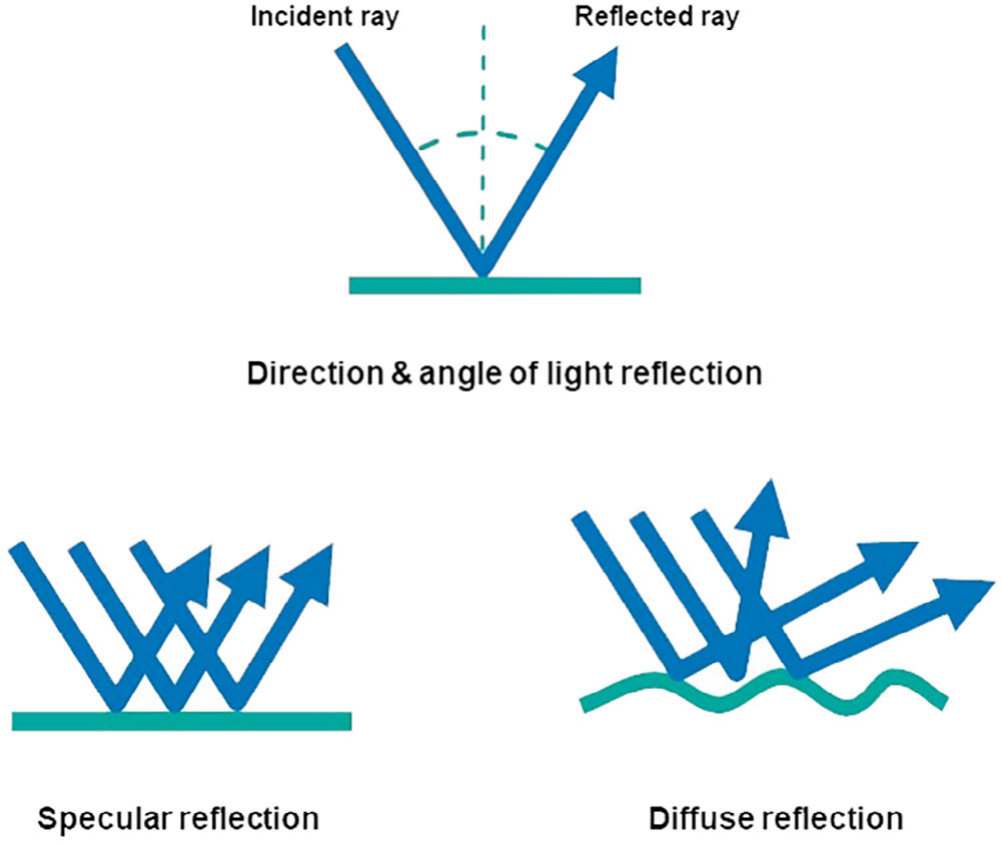
Smooth surfaces reflect light uniformly, resulting in clear and defined reflections, giving them a shiny appearance. In contrast, rough surfaces scatter light in multiple directions due to their uneven texture, leading to diffused reflections and a matte appearance.

The mechanism by which this smoothing and wrinkle‐reducing effect is achieved involves subdermal filling that induces skin stretching, thereby increasing the density of dermal and subdermal tissues. This process results in an indirect blockage at the musculocutaneous junction, effectively smoothing out wrinkles. The technique and plane of soft filler injection beneath the skin are strategically varied to optimize this effect, as illustrated in Figure 6. Additionally, the impact of SSRT treatment in volumizing and refining the skin's surface extends beyond merely altering the face's contour or shape. When one observes an object's surface, the tactile sensation upon contact is integrated with the visual texture perceived, fostering a sensory experience that allows for the mental recreation of the surface texture without direct touch. This enhancement in visual texture is significant. Another critical aspect of visual texture is the degree of light reflection off the surface, which affects our perception of the object's gloss, transparency, depth, thickness, and wrinkling based on the surface structure. For instance, when light strikes the facial skin, it scatters in various directions, yet the incident and reflected rays form specific angles as depicted in Figure 7. A smooth skin surface yields a more uniform reflection angle, reducing shadows and enhancing the visual texture through specular reflection. Conversely, on rough, uneven skin surfaces characterized by large pores or pronounced wrinkles, diffuse reflection predominates, resulting in shadowing and a mottled appearance (Figure 7).^30^, ^31^

**FIGURE 8:**
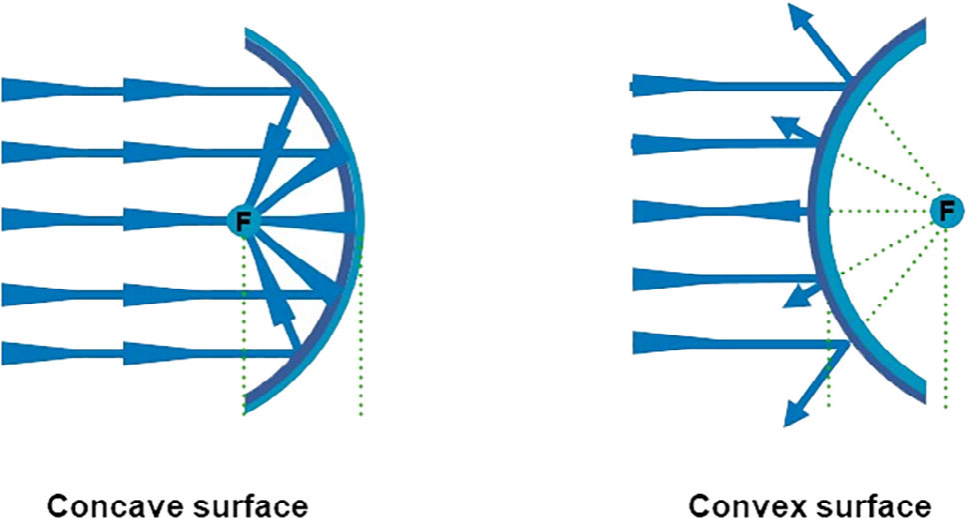
Light reflection on skin varies with its contour: smooth surfaces reflect light evenly, enhancing luminosity and giving a youthful appearance, while uneven surfaces like wrinkles or scars scatter light, highlighting imperfections and making the skin appear duller.

Additionally, light reflection is influenced by the skin's surface contours. If light strikes a concave surface, as depicted on the left side of Figure 8, it reflects inward toward the focus (F), diminishing light scatter and the gloss effect. Conversely, light impacting a convex skin surface, as shown on the right side of Figure 8, scatters outward from the focus (F), enhancing light dispersion and producing a gloss effect, often described as a radiant face. This is a characteristic commonly observed in youthful faces with ample volume and well‐defined skin contours, and achieving this effect is a primary goal of SSRT treatment.

**FIGURE 9:**
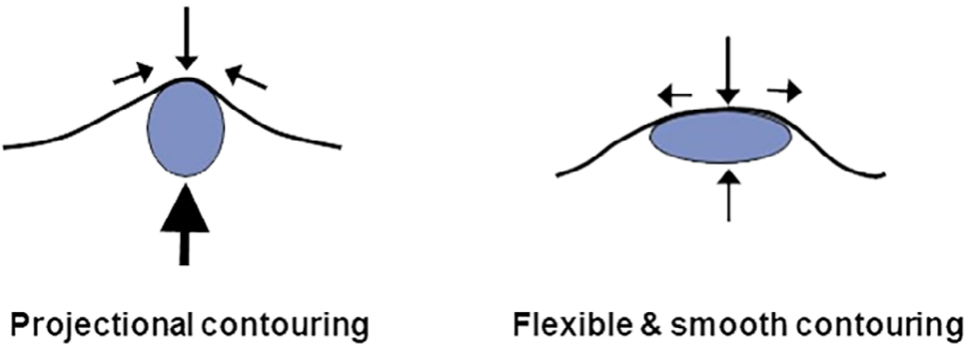
Two types of volumizing HA fillers are tailored for specific facial contouring based on the skin and SMAS layer characteristics: High G* fillers and Medium G* fillers with good cohesion. High G* fillers are ideal for areas with thick and tight skin, providing pronounced, projectional contouring suitable for regions requiring substantial volume and lift. In contrast, Medium G* fillers are better suited for thinner, softer skin and a more mobile SMAS layer, offering flexible and smooth contouring that allows for natural facial movements while still enhancing and smoothing the facial features. Each type supports distinct aesthetic goals, either by adding significant definition or by subtly enhancing natural facial expressions.

When undertaking dual plane filler injections, the viscoelastic properties of the firm filler intended for the subSMAS layer are critical. In regions requiring precise contour maintenance regardless of facial movements, such as the nose, chin tip, jawline, zygomatic central point, and nasal bone depression, a filler with high elasticity should be utilized to avoid shape distortion from surrounding muscle activity and to preserve the original, clear contours.^32^ Conversely, in areas prone to frequent movement, such as nasolabial folds, corners of the mouth, front cheeks, and perioral cheek regions, using a highly elastic filler may lead to discomfort and unnatural expressions. Thus, a filler with appropriate viscoelasticity and cohesiveness is essential, capable of adapting during movement and reverting to its original form while maintaining a soft, non‐angular volume (Figure 9).

**FIGURE 10:**
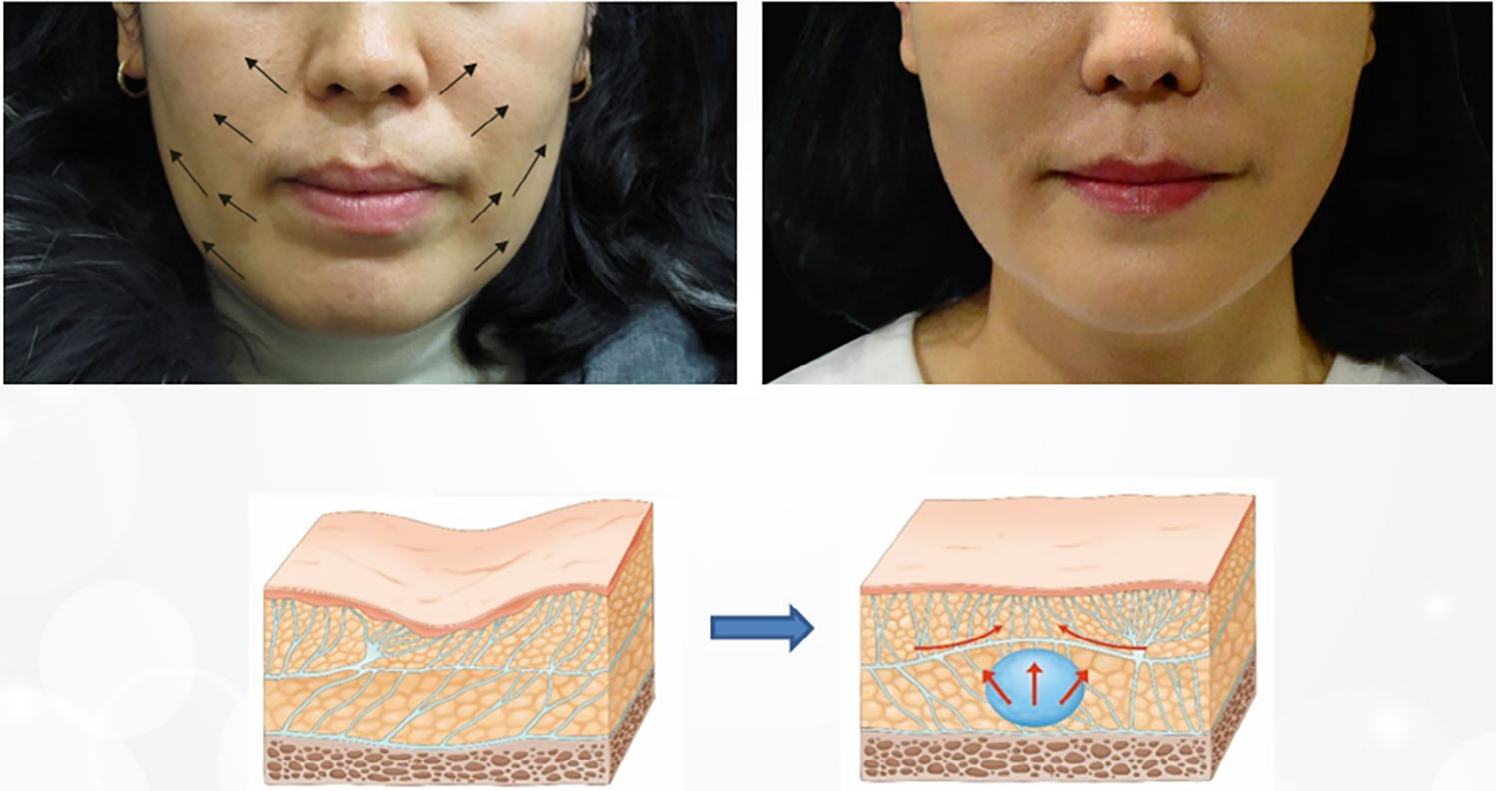
SSRT, is a skin remodeling technique that targets the SMAS layer. It's designed to improve facial contours by releasing the fibrous attachments within the SMAS, leading to enhanced skin tightening and lifting. This approach effectively rejuvenates the face by repositioning deeper tissues, which helps to smooth and refine the overall facial structure.

For regions requiring accommodation for facial movements, such as the midfacial nasolabial fold area and the lower facial perioral area, the use of a filler with optimal viscoelasticity and cohesiveness is essential for achieving a slimming effect while preserving appropriate volume.^33^ The cascading impact of this approach on adjacent areas can be substantiated through clinical examples. For example, filler injections in the zygomatic area influence the nasolabial folds, injections in the nasolabial folds impact the upper lip, and injections in the front cheek area alter the appearance of the lower lip.^34^, ^35^, ^36^, ^37^ Similarly, filler placement in the prejowl and lateral cheek areas respectively affect the chin tip and jawline, demonstrating interconnected lifting effects (Figure 10).

**FIGURE 11:**
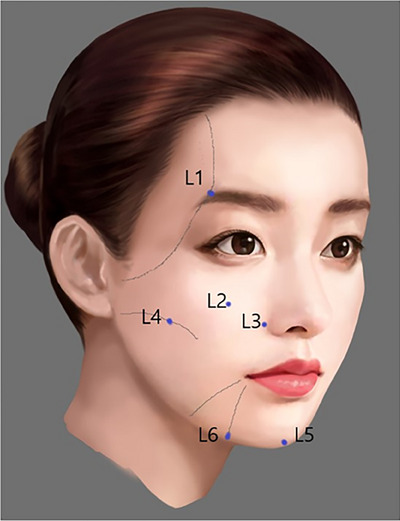
The lifting technique of SSRT involves precise filler injections across several facial sites using very high G* filler to enhance and define features. In the upper lateral eyebrow region (L1), specifically at the temporal ligament adhesion, 0.1 mL of filler is used to create a subtle lift. For the Indian band region (L2), where the zygomatico‐cutaneous ligaments are located on the anterior malar region, 0.2 mL of filler helps accentuate this area. Similarly, 0.2 mL is applied to the nasolabial band region (L3), targeting the medial maxillary ligament for a more pronounced effect. The anterior lateral cheek hollow (L4), which involves the masseteric‐retaining ligaments under the malar eminence, also receives 0.2 mL to enhance the cheek's contour. The chin region (L5), associated with the mental ligament, and the lateral border of the chin (L6) near the medial mandibular ligament each receive 0.2 mL and 0.1 mL respectively, refining and defining the lower facial contours effectively.

**FIGURE 12:**
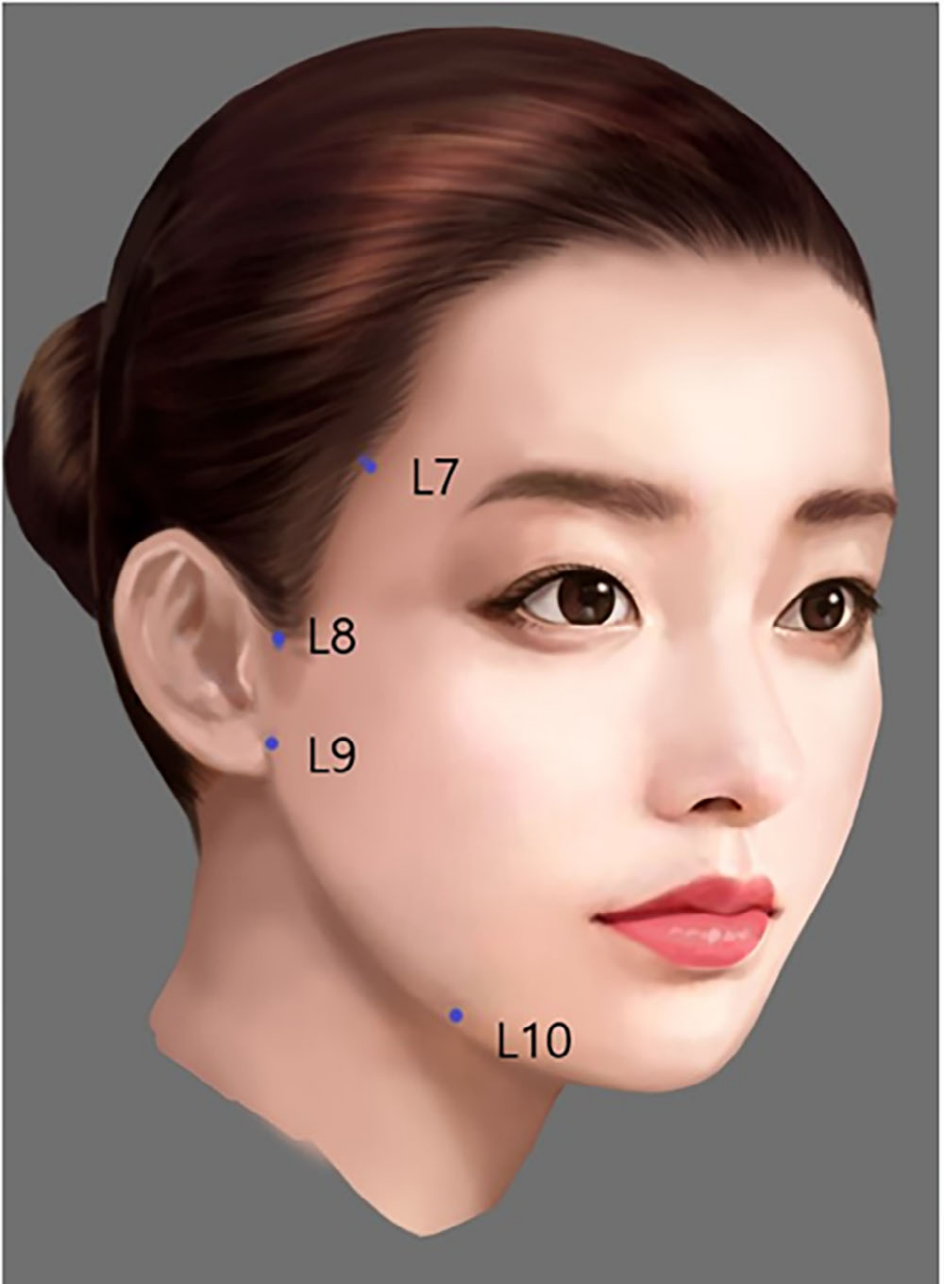
The SSRT lifting technique includes targeted filler injections in specific facial areas to enhance and define features effectively: 0.1 cc of very high G* filler is administered at the temple hairline region (L7), affecting the temporal septum for subtle enhancements. The upper zygomatic arch region (L8), associated with the temporal fascia, and the lower zygomatic arch region (L9), where the zygomatic ligaments are located, each receive 0.1 cc to accentuate the cheekbones and frame the face. Additionally, the labiomandibular fold (L10), influenced by the lateral mandibular ligament, is treated with 0.1 cc to smooth out marionette lines and sharpen the jawline, completing the contouring process with precision.

In practice, these techniques are often synergistically combined, though SSRT treatments are primarily categorized into three distinct approaches based on their specific objectives. The lifting technique, which employs a minimal amount of very firm filler strategically placed within each facial area, is designed to induce a natural lifting effect. The contouring technique focuses on sculpting an attractive facial silhouette and adding necessary volume. Lastly, the stretching technique involves the subcutaneous injection of soft fillers to effectively tighten the skin and smooth out fine lines and wrinkles, thereby enhancing the overall texture and appearance of the skin (Figures 11 and 12).

**FIGURE 13:**
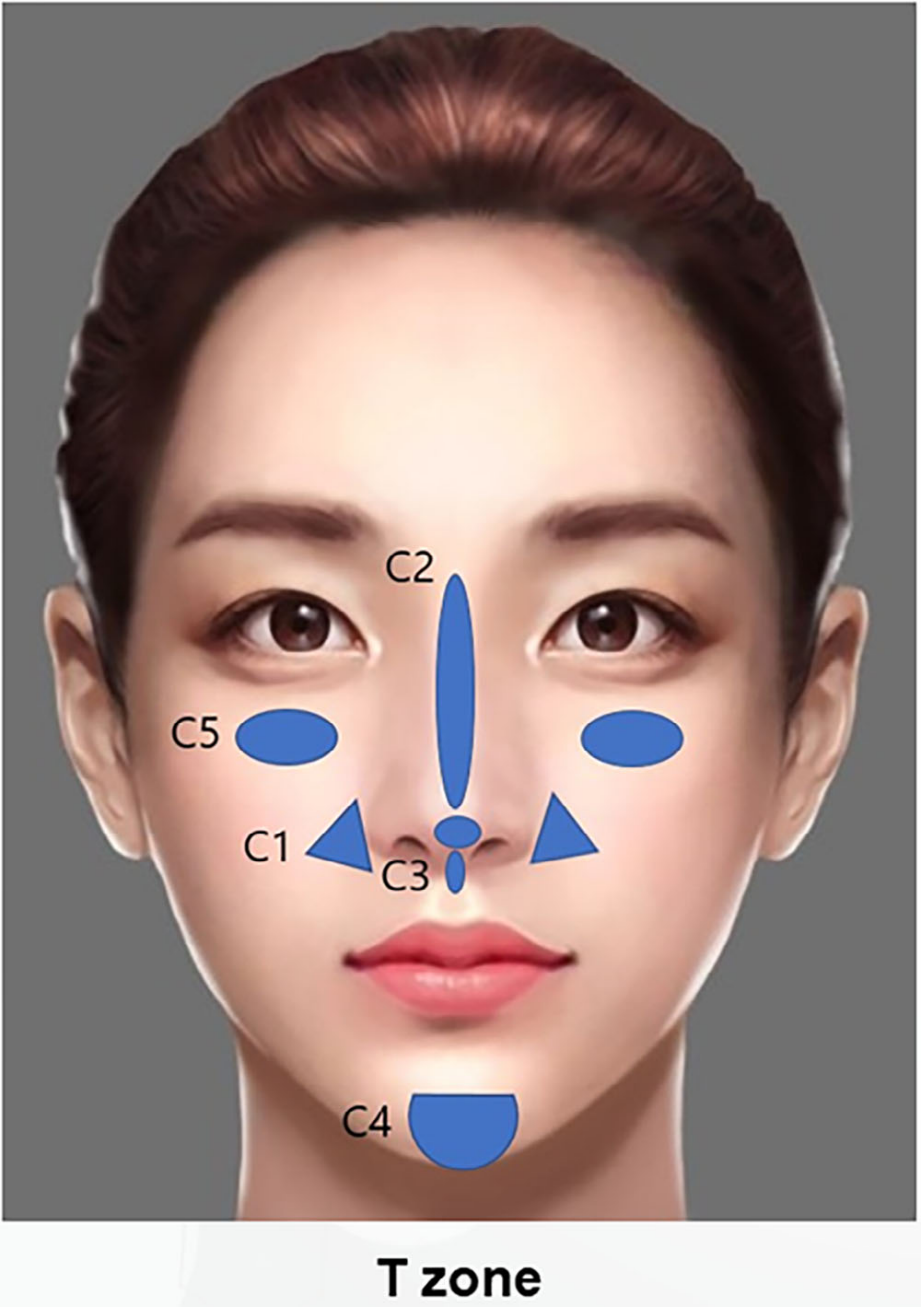
The contouring technique of SSRT includes precise filler applications at various facial sites to enhance and define the facial structure. In the nasolabial folds, particularly affected by paranasal depression (C1), 0.5 to 0.7 mL of high to medium G* filler is injected on each side for volume restoration and contour smoothing. The nasal dorsum (C2) receives a 0.5 mL injection of very high G* filler to refine and elevate the bridge of the nose. For more detailed shaping, the nasal tip region (C3) is enhanced with 0.1 mL of high G* filler, and the columellar region is further defined with 0.2 mL of very high to high G* filler. The chin region (C4) is contoured with 0.5–0.7 mL of very high G* filler to improve its projection and symmetry. Finally, the anterior malar region (C5) is sculpted with 0.4–0.5 mL of high to medium G* filler on each side, emphasizing the cheekbones and enhancing facial harmony.

**FIGURE 14:**
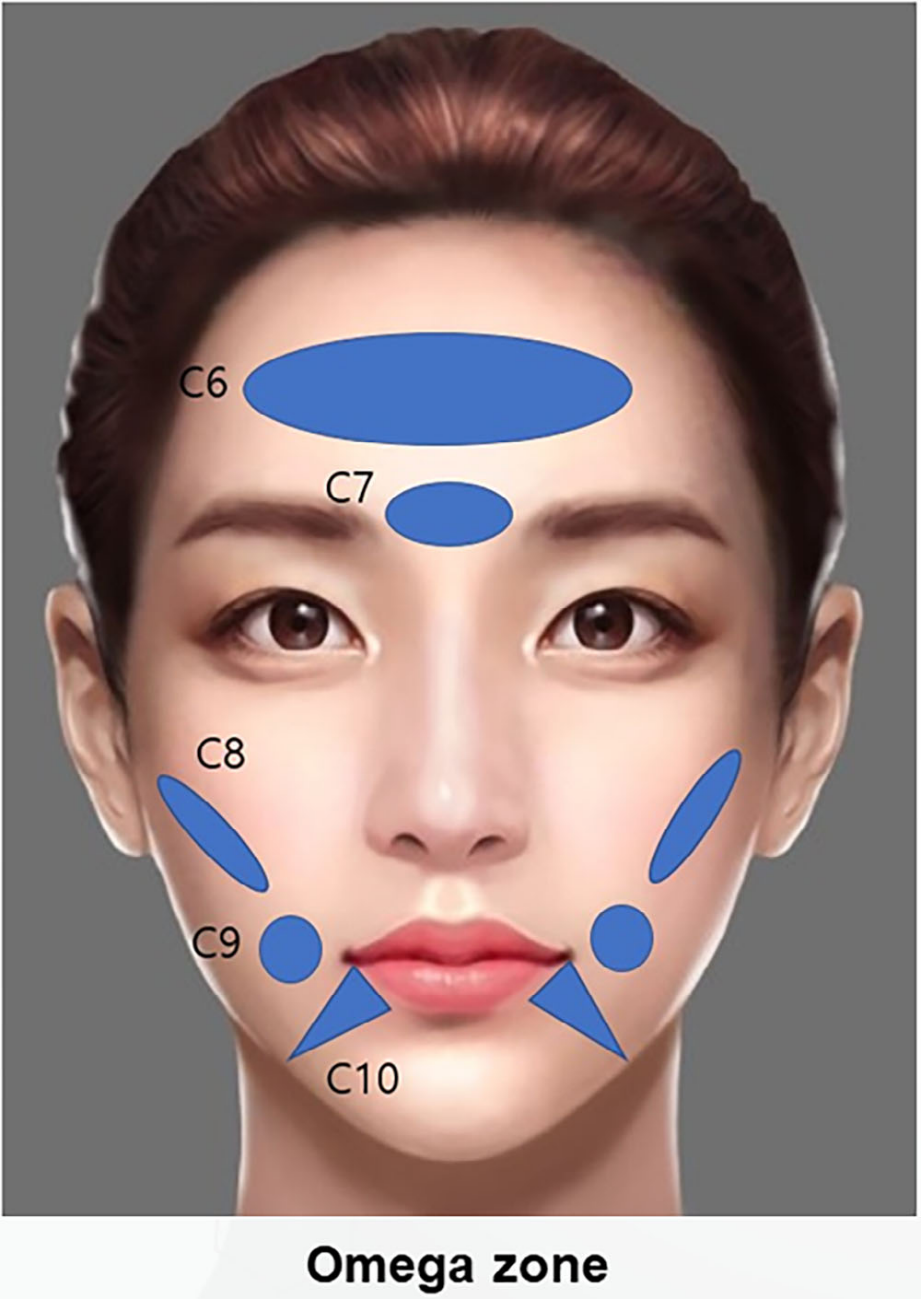
The SSRT contouring technique employs strategic filler injections across various facial areas to enhance and define contours effectively: The forehead region (C6) is enhanced with 0.7 mL of high to medium G* filler to smooth and volumize. In the glabellar region (C7), between the eyebrows, 0.2–0.3 mL of high G* filler minimizes frown lines and refines texture. For more pronounced cheek definition, the lateral cheek hollows (C8) receive a total of 1.0 mL (0.5 mL each side) of very high to high G* filler, emphasizing the contours of the face. The buccal cheek region (C9) is subtly augmented with 0.2 mL of medium G* filler on each side to provide fuller cheeks. Additionally, the deep labiomandibular folds (C10), which contribute to an aged appearance, are softened with 0.2 mL of high to medium G* filler on each side, smoothing the transition from the jawline to the chin.

**FIGURE 15:**
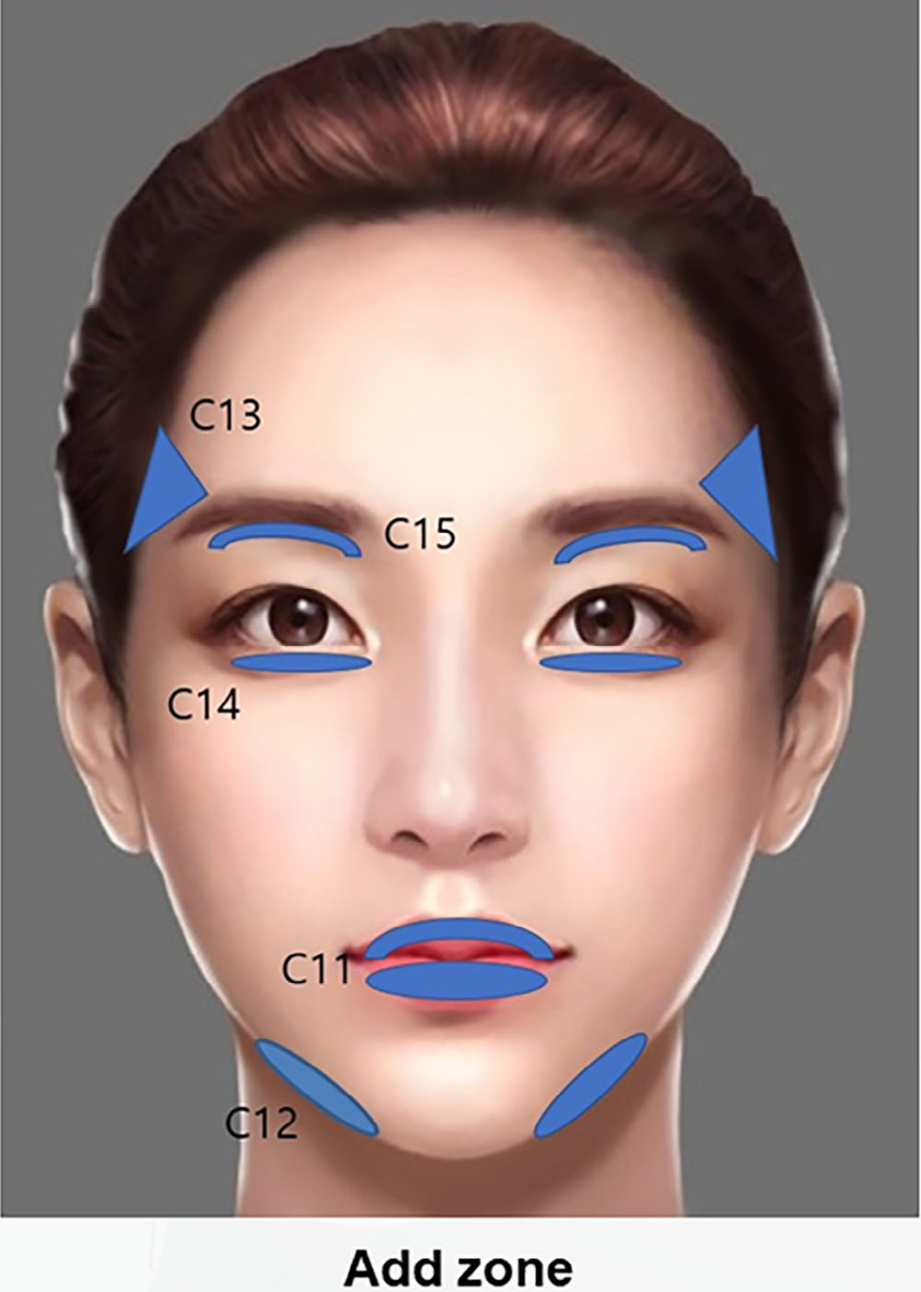
The SSRT contouring technique meticulously enhances various facial features with specific filler injections: The upper lip (C11) is subtly volumized with 0.3 mL of high to medium G* filler, and the lower lip receives a more generous 0.5 mL to further define its shape. To sharpen the jawline, the mandible border line (C12) is contoured with a total of 0.6 mL (0.3 mL on each side) of very high to high G* filler. Temple depressions (C13) are filled with 1.0 mL (0.5 mL each side) of very high to high G* filler to restore volume and smoothness. The lower eyelid charming roll (C14) is enhanced with 0.6 mL (0.3 mL each side) of medium to low G* filler, contributing to a refreshed and youthful look. Additionally, sunken upper eyelids (C15) are rejuvenated with 0.6–0.8 mL (0.3–0.4 mL each side) of medium to low G* filler, helping to alleviate the appearance of hollows and restoring a more alert and vibrant eye appearance. These injections are strategically placed to ensure natural‐looking, harmonious facial enhancement.

**FIGURE 16:**
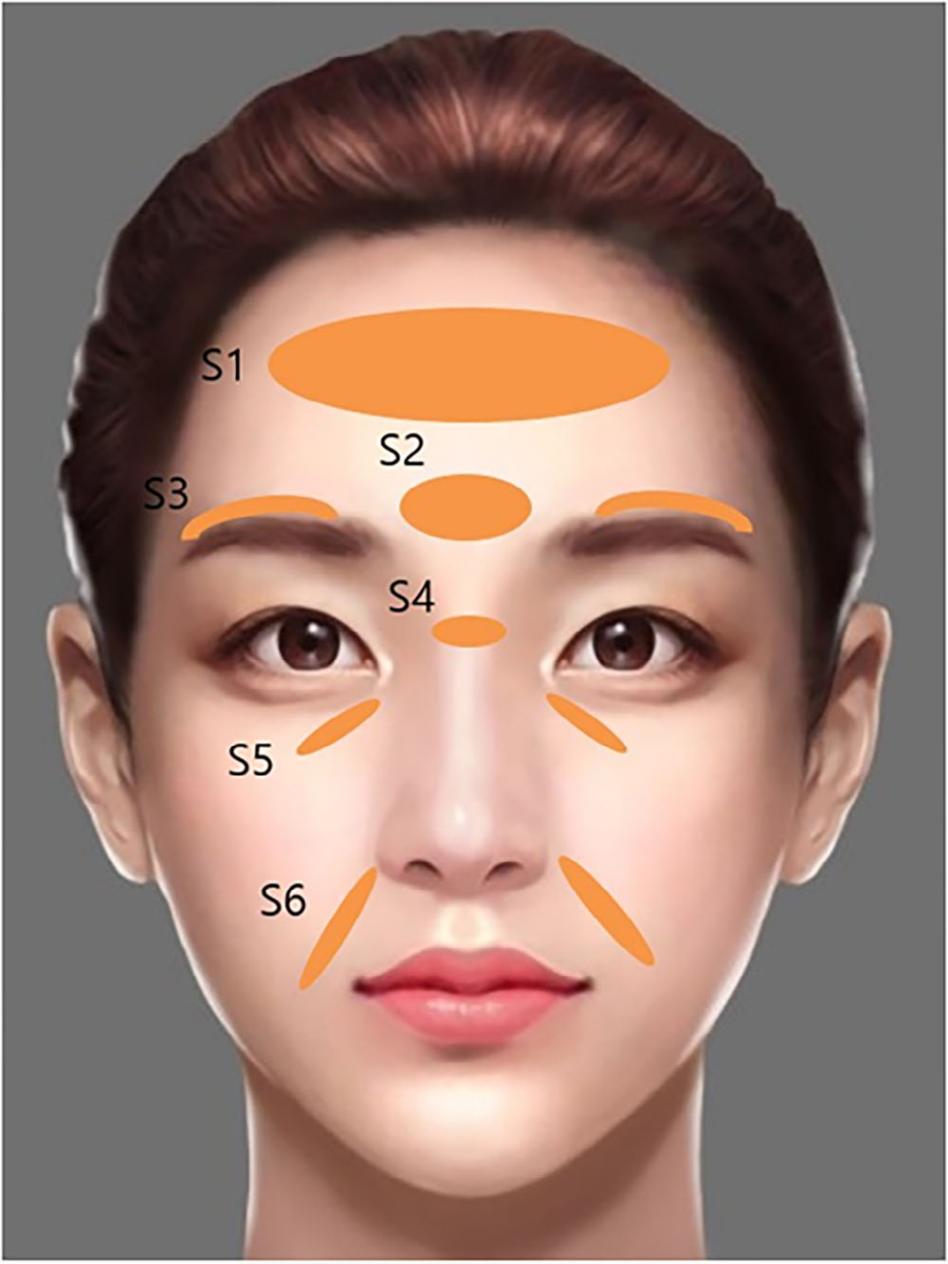
The stretching technique of SSRT targets various areas of the face to alleviate signs of aging through strategically placed filler injections. For forehead wrinkle lines (S1), 0.3 mL of low G* filler is used to smooth out creases, creating a more youthful appearance. In the glabellar region (S2), characterized by frown lines, 0.2 mL of low G* filler is applied to reduce the depth of wrinkles. The upper eyebrow wrinkled region (S3) receives a total of 0.4 mL (0.2 mL each side) of low G* filler to soften expression lines and lift the brows subtly. For the nasal frowning region (S4), a small dose of 0.1 mL of high to low G* filler is injected to diminish the severity of frown lines. The tear trough deformity (S5), which can create a tired appearance, is treated with 0.4 mL (0.2 mL each side) of low to very low G* filler to restore volume and reduce shadowing under the eyes. Similarly, nasolabial creases (S6) receive 0.4 mL (0.2 mL each side) of low to very low G* filler to smooth out smile lines, enhancing the overall facial expression. These injections are carefully administered to ensure natural‐looking results and improved skin elasticity.

**FIGURE 17:**
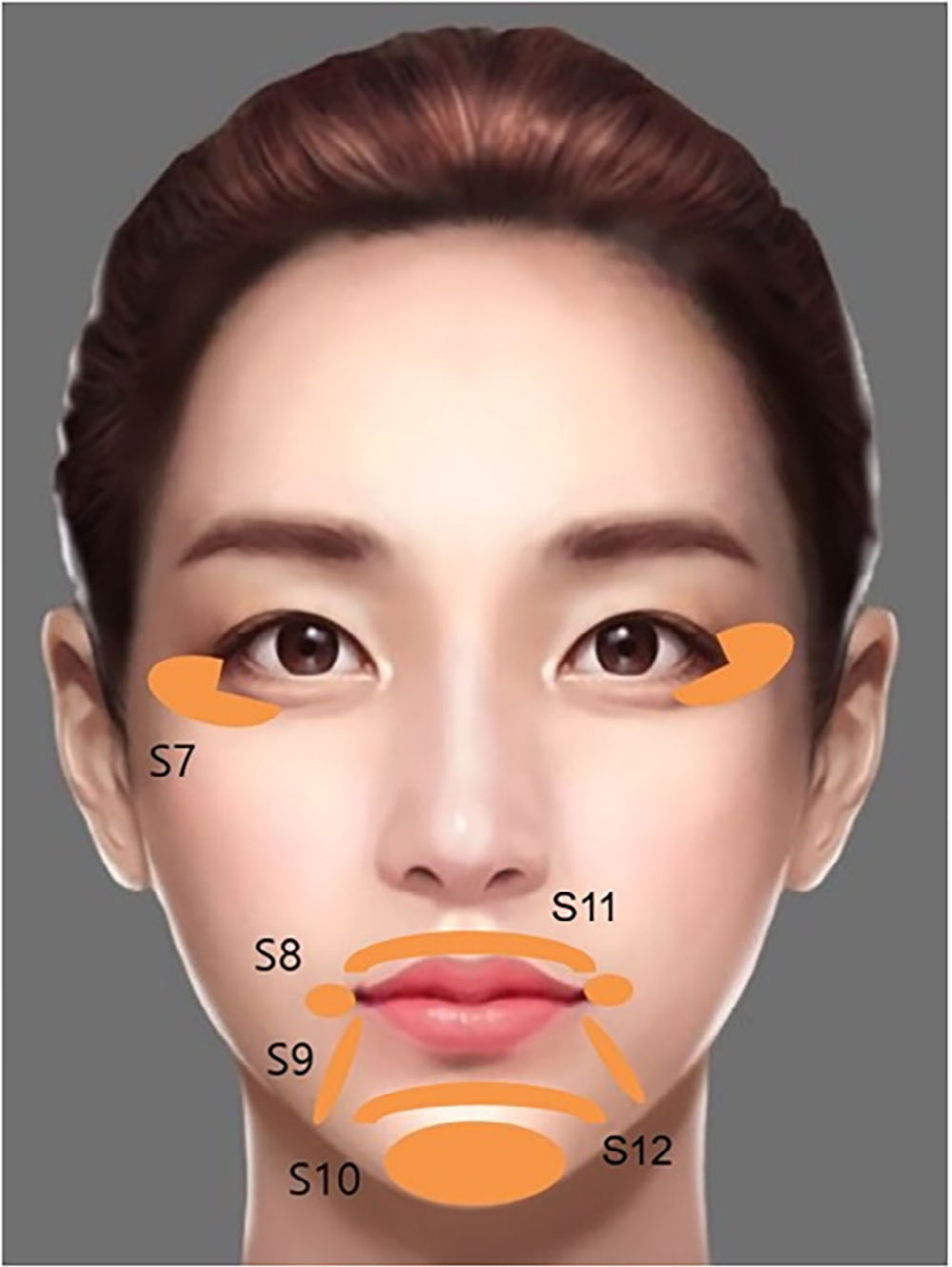
The SSRT stretching technique addresses various facial concerns through targeted filler injections: Crow's feet lines (S7) are softened with a total of 0.2 mL (0.1 mL each side) of very low G* filler. The mouth corner drooping region (S8) receives 0.2 mL (0.1 mL each side) of low G* filler to lift and reduce sagging. Marionette lines (S9) are smoothed with 0.4 mL (0.2 mL each side) of low to very low G* filler, enhancing the lower face. Chin dimpling region (S10) is corrected with 0.2–0.3 mL of medium to low G* filler for a smoother chin texture. Vertical wrinkles (S11) on the upper lip are treated with 0.2 mL of very low G* filler to diminish their appearance, and the labiomental crease (S12) between the lower lip and chin is refined with 0.2–0.3 mL of low G* filler, improving the facial profile. This comprehensive approach ensures that each area is enhanced while maintaining the natural dynamics of facial expressions.

The fundamental mechanisms of each SSRT technique merit further exploration. The lifting technique leverages changes in the lifting vector observed when a person lies down, utilizing subSMAS filler injections in areas with robust ligamentous tissues. This densely packs the tissue, pulling adjacent looser tissues upward, thereby creating a natural lifting effect, as illustrated in Figures 11 and 12. Conversely, the contouring technique is inspired by the dynamic volumetric alterations observed in a face when smiling. This method aims to replicate a smiling visage by sculpting facial contours and enhancing key features such as the nose, chin tip, jawline, and zygomatic central point, while filling in recessed areas to even out the facial surface, depicted in Figures 13, 14, 15. The stretching technique, shown in Figures 16 and 17, is designed to tighten the skin and mitigate wrinkles by evenly stretching the skin surface, thus enhancing the overall texture and appearance.

The typical facial areas targeted by each technique and the necessary properties and volumes of fillers have been delineated.^4^ Nonetheless, the selection of fillers during treatment should be predicated on their physical properties and customized to accommodate the individual skin and soft tissue conditions of each patient, as well as their specific aesthetic objectives. This tailored approach ensures that the fillers not only enhance the desired areas effectively but also harmonize with the patient's natural facial dynamics and structural characteristics, optimizing both the visual and functional outcomes of the treatment.

### 1.2 Myomodulation theory

Dr. De Maio's concept of myomodulation originates from Brazil and posits that manipulating facial expression muscles via filler injections can foster a lifting effect.^6^, ^38^ This technique leverages the dynamics between levator muscles, which assist in forming a smile and are thus termed synergists, and depressor muscles, which facilitate frowning and are viewed as antagonists. Dr. De Maio proposes that strategically using HA fillers to adjust muscle activities can significantly enhance facial aesthetics by restoring balance to abnormal muscle movements.

In his comprehensive discourse on myomodulation, Dr. De Maio underscores its importance and elucidates the methodology with three core principles: the Length‐tension relationship, the Muscle pulley & lever system, and the Functional muscle group. These principles were thoroughly discussed in his 2018 publication, which clarifies the differential impacts of fillers when administered above versus below the muscles, illustrating the biomechanical foundations of myomodulation in aesthetic medicine.^38^

### 1.3 True lift

Taiwanese plastic surgeon Peter Hwang has developed the True Lift method, a technique premised on the notion that facial aging is often characterized by the loosening of supportive ligaments, leading to sagging skin and soft tissues.^39^ According to this method, strategically injecting fillers beneath these key ligaments can bolster the sagging structures, effectively pulling and lifting the skin to achieve a youthful appearance. This method specifically targets six primary retaining ligaments with firm, consistent fillers: the zygomatic ligaments above the zygomatic arch, the platysma‐auricular ligament below the ear lobule, the masseteric‐cutaneous ligaments along the anterior boundary of the masseter muscle, the orbital retaining ligament encircling the orbital rim, the maxillary ligament near the nasolabial folds, and the mandibular ligament along the marionette line area of the jawline.

**FIGURE 18:**
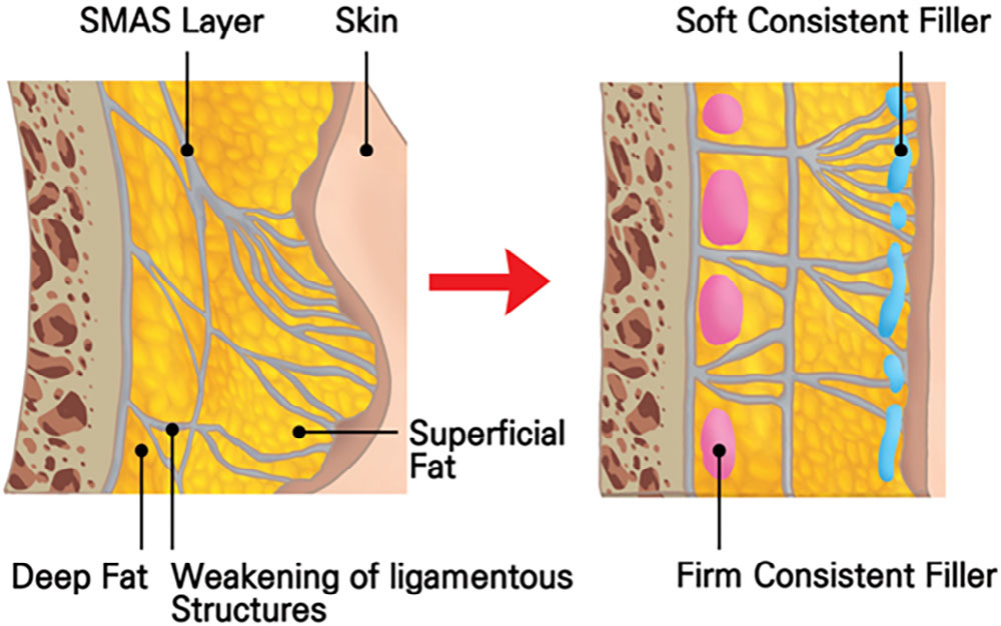
Natural lifting is achieved by injecting firm consistent fillers into the subSMAS layer, which increases the density of ligamentous tissues. This technique enhances facial support structures, offering a lifted and youthful appearance while maintaining a natural look by reinforcing the underlying anatomical features.

The True Lift shares conceptual similarities with the SSRT by focusing on specific facial ligaments to facilitate a lifting effect. However, Hwang's theory refines the notion that ligament tissues rigidly “stand up” upon filler injection. Instead, it suggests a more nuanced view where the injection of fillers increases the density beneath these ligaments, thus firming the area. This technique is not restricted to the six primary areas; it can be applied wherever strong ligamentous tissues support the skin, potentially producing lifting effects. Effective application of this method relies heavily on a comprehensive anatomical understanding of facial ligamentous structures, ensuring targeted and successful lifting outcomes (as depicted in Figure 18).

### 1.4 True myomodulation for the latest insights

The human face is densely populated with over 17 000 sensory receptors embedded within its musculature, and although the intrinsic sensory system of facial muscles is not fully elucidated, these receptors are broadly categorized into slow adapting (SA) and rapidly adapting (RA) types. Slow adapting receptors primarily transmit signals via the autonomic nervous system (ANS), modulating both sympathetic and parasympathetic responses crucial for facial expressions. They serve a dual function: reducing sympathetic tone and emotional arousal—which dampens activity in the depressor muscles and the frontalis muscle on the forehead—and concurrently activating the elevator muscles, which are involved in parasympathetic responses that foster emotional relaxation.

This nuanced interplay of autonomic responses can precipitate distinct facial movements. Activation of parasympathetic responses typically results in the contraction of elevator muscles around the mouth, eliciting a natural smile, while simultaneously reducing activity in the forehead muscles, which are otherwise engaged during sympathetic excitation. This modulation is mediated by specific mechanoreceptors in the muscles that respond to ANS signals, influencing all facial muscles comprehensively. For instance, strategic filler injections near the cheek muscles can amplify the contraction of elevator muscles, thereby elevating the corners of the mouth. Conversely, placing fillers beneath the depressor anguli oris (DAO) muscle can inhibit depressor muscles, averting the downturn of mouth corners. Similarly, administering injections below the forehead muscles can inhibit their contraction, thereby smoothing forehead wrinkles and enhancing aesthetic outcomes.

**FIGURE 19:**
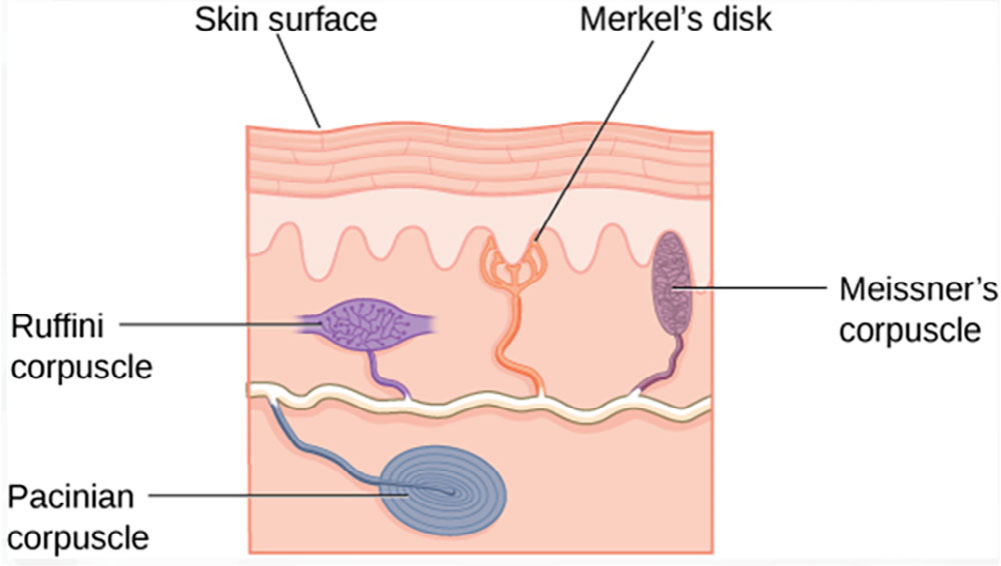
Ruffini‐like corpuscles in facial muscles for mechanoreceptor filler hypothesis.

Recent immunohistochemical studies have revealed the presence of Ruffini‐like corpuscles within the zygomaticus major muscle, akin to those found in the skin.^40^ This discovery implies that mimetic muscle mechanoreceptors potentially modulate muscle activity in response to filler injections, corroborating the mechanoreceptor filler hypothesis. Should this hypothesis prove accurate, fillers would not only alter facial architecture but also enhance emotional expressions, thereby facilitating genuine myomodulation, as illustrated in Figure 19.^41^

Moreover, the administration of small particle HA fillers into the dermis significantly boosts skin volume, hydration, and fibroblast activity.^42^ This, in turn, increases collagen production and antioxidant activities, which enhance skin elasticity and texture. It is posited that stimulating the soft tissue with HA fillers initiates immediate cell contraction followed by tissue remodeling, through the activation and proliferation of fibroblasts. Additionally, employing soft tissue HA volume fillers for facial volumization impacts subcutaneous white adipose tissue (sWAT), activating adipose‐derived stem cells (ADSCs) and promoting the expansion of mature adipocytes.^42^, ^43^, ^44^ This results in sustained volumization and tissue firming via hyperplasia and hypertrophy of ADSCs and mature adipocytes, although further research is essential to comprehensively understand these mechanisms.

## 2 DISCUSSION

In the evolving landscape of facial aesthetics, the SSRT represents a significant advancement in achieving not only volumization but also a natural lifting effect through strategic filler injections. This technique hinges on a precise understanding of the anatomical and dynamic properties of facial soft tissues, particularly how they interact under different expressions and orientations. Traditional approaches often focus solely on filling wrinkles or replacing lost volume, but SSRT aims to mimic the natural lift seen when smiling, using both firm and soft fillers to affect the SMAS layer and underlying structures.

The clinical application of SSRT involves a nuanced use of fillers of varying consistencies—firm for deeper structural support and soft for superficial smoothing—to sculpt a naturally youthful and dynamic appearance. By strategically increasing tissue density beneath the SMAS, the technique leverages the natural tensile properties of facial connective tissues to induce a lifting effect, pulling sagging areas upward and outward, mimicking the effect of facial expressions like smiling. This approach not only enhances facial symmetry and contours but also respects the natural movement, avoiding the stiffness often associated with overfilled faces.

Furthermore, SSRT considers the gravitational effects on facial tissues, providing insights into how facial contours can be optimized by simulating the appearance of lying down, where gravity pulls the tissues differently, suggesting a potential for a transformative ‘lift’ when upright. This concept is visualized through advanced imaging techniques that track the shifting of tissues, offering a compelling visual proof of concept for SSRT's effectiveness.

As we compare SSRT with other globally recognized methods like the True Lift and Myomodulation, it becomes evident that each technique has its merits depending on the specific needs and anatomical considerations of the patient. SSRT, in particular, excels in creating a harmonious balance between volume enhancement and dynamic facial expression, achieving a visibly lifted but natural appearance without the risks associated with excessive filler use.

By integrating SSRT into clinical practice, practitioners can offer a more tailored aesthetic solution that goes beyond traditional filling techniques, addressing the patient's aesthetic concerns with a holistic approach that considers both the static and dynamic aspects of the face. This method not only enhances the immediate visual appeal but also promotes a longer‐lasting, natural‐looking result, aligning with the modern ideals of subtle and effective cosmetic intervention.

## CONFLICT OF INTEREST STATEMENT

The authors declared no potential conflicts of interest with respect to the research, authorship, and publication of this article. This study was conducted in compliance with the principles set forth in the Declaration of Helsinki.

## Data Availability

The data that support the findings of this study are available from the corresponding author upon reasonable request.
